# Personalized Therapy Against Preeclampsia by Replenishing Placental Protein 13 (PP13) Targeted to Patients With Impaired PP13 Molecule or Function

**DOI:** 10.1016/j.csbj.2017.09.002

**Published:** 2017-09-22

**Authors:** Hamutal Meiri, George Osol, Irene Cetin, Sveinbjörn Gizurarson, Berthold Huppertz

**Affiliations:** a Hy Laboratories, Rehovot, and TeleMarpe, Tel Aviv, Israel; b Department of Obstetrics, Gynecology and Reproductive Sciences, University of Vermont, Burlington, VT, USA; c Department of Obstetrics and Gynecology, University of Milano, Italy; d Department of Mother and Child, Hospital Luigi Sacco, and Center for Fetal Research “Giorgio Pardi”, Milano, Italy; e Faculty of Pharmaceutical Sciences, School of Health Science, University of Iceland, Reykjavik, Iceland; f Institute of Cell Biology, Histology and Embryology & Biobank Graz, Medical University of Graz, Graz, Austria

**Keywords:** Preeclampsia, Galectins, LGALS13, Polymorphism, Placenta, Gene expression, Promoter regulation, Immune tolerance, Endothelial layer, Signaling pathways

## Abstract

Hypertensive disorders affect about one third of all people aged 20 and above, and are treated with anti-hypertensive drugs. Preeclampsia (PE) is one form of such disorders that only develops during pregnancy. It affects ten million pregnant women globally and additionally causes fetal loss and major newborn disabilities. The syndrome's origin is multifactorial, and anti-hypertensive drugs are ineffective in treating it. Biomarkers are helpful for predict its development. Generic drugs, such as low dose aspirin, were proven effective in preventing preterm PE. However, it does not cure the majority of cases and many studies are underway for fighting PE with extended use of additional generic drugs, or through new drug development programs.

This review focuses on placental protein 13 (PP13). This protein is only expressed in the placenta. Impaired PP13 DNA structure and/or its reduced mRNA expression leads to lower blood PP13 level that predict a higher risk of developing PE. Two polymorphic PP13 variants have been identified: (1) The promoter PP13 variant with an “A/A” genotype in the -98 position (versus “A/C” or “C/C”). Having the “A/A” genotype is coupled to lower PP13 expression, mainly during placental syncytiotrophoblast differentiation and, if associated with obesity and history of previous preeclampsia, it accurately predicts higher risk for developing the disorder. (2) A thymidine deletion at position 221 causes a frame shift in the open reading frame, and the formation of an early stop codon resulting in the formation of DelT_221_, a truncated variant of PP13. In pregnant rodents, both short- and long- term replenishment of PP13 causes reversible hypotension and vasodilation of uterine vessels. Long-term exposure is also accompanied by the development of larger placentas and newborns. Also, only w/t PP13 is capable of inducing leukocyte apoptosis, providing maternal immune tolerance to pregnancy.

Based on published data, we propose a targeted PP13 therapy to fight PE, and consider the design and conduct of animal studies to explore this hypothesis. Accordingly, a new targeted therapy can be implemented in humans combining prediction and prevention.

## 1 Preeclampsia: A Unique Form of Hypertension in Pregnancy With the Need for an Orphan Drug Approach to Cure Preeclampsia — The PP13 Drug Development Approach

### 1.1 Hypertension and its Consequences

Hypertension - defined as blood pressure (BP) above 140 systolic over 90 diastolic mmHg -is a growing global epidemic. According to the 2011 World Health Organization (WHO) report, the number of people living with hypertension (high BP) is predicted to be 1.56 billion worldwide by the year 2025 [1]. Hypertension affects about a third of all people aged 20 and above, and is treated with anti-hypertensive drugs [2], [3]. According to the 2010 World Health Statistics there are more people in Europe and the USA die from hypertension-related cardiovascular diseases (CVDs) than from the next three deadliest diseases combined. Thus, the control of hypertension has become a key priority worldwide [4], [5] and is accompanied with major investment in drug research.

The increased prevalence of hypertension is attributed to the growing epidemics of obesity and metabolic disorders, factors associated with lifestyle (e.g., lack of physical activity, alcohol and tobacco use, and a high consumption of sugar and salt in processed food), family history, and anxiety [6]. Chronic high BP leads to blood vessel narrowing induced by altered shear forces of the blood flow against the arterial vessel wall [7]. Its incidence steadily increases with age as smaller arteries become stiffer and narrower, while plaque formation in large vessels is accelerated, and leads to atherosclerosis, favoring the development of CVD [8], [9]. Additional risk factors include diabetes, kidney diseases, pheochromocytoma, Cushing syndrome, congenital adrenal hyperplasia, hyperthyroidism, black ethnicity and sleep apnea [10], [11], [12].

### 1.2 Preeclampsia as a Specific Type of Hypertension

Preeclampsia (PE) is a unique form of hypertension that is specific to pregnant women. Its clinical symptoms develop in the midst of pregnancy in previously normotensive women, and it is accompanied by protein loss in the urine (proteinuria). There are multiple complications in other organs (liver, blood, lung, and eyes) with varying severity of symptoms. The disorder can be exacerbated to convulsion and stroke (eclampsia). Only delivery and removal of the placenta stops the disorder [13], [14], [15]. The healthcare burden of PE is considerable, with estimated annual global healthcare costs of $3 billion [16].

PE is a major life threatening syndrome for pregnant women, and its frequency is on the rise due to increased rates of obesity and pregnancy at advanced maternal age [17], [18], [19]. Today there is no drug that is generally accepted for treating all cases of the disorder; neither is it clear when to treat. The preeclampsia foundation (*www.preeclampsiafoundation.com*) has indicated that each year about 76,000 maternal lives are lost due to PE. Current practice is to deliver the baby to prevent the risk of developing eclampsia, a final stage of the disorder associated with convulsions, stroke and death. Hence, if the disorder develops early, the baby has to be delivered prematurely, and there are around 300,000–500,000 life losses and major handicaps of newborns associated with PE. These tragedies massively affect pregnant women and their families.

Regrettably, there are impediments in the effort to develop effective drug prevention programs to stop the devastating effects of PE on pregnant women and their babies. (1) Only 0.5% or less of the total health research budget in Europe is dedicated to fight PE, and the investments elsewhere are also limited [20]. (2) Drug companies do not view this syndrome as a target for developing the next “block buster” [21], [22], [23], [24]. (3) On the contrary, most companies are worried of entering the pregnancy field as they have concerns of potential teratogenic effects (“the thalidomide impact”) [25] or of other long-term reproductive effects (the “DES” story) [26]. As a result, there is almost a complete stifling of the development of new therapeutics against PE. The field remains almost completely in the hands of medical and academic leaders, and of a few small companies. Since a new drug development requires large resources, it is no wonder that the progress is slow.

Today, PE management is performed by close surveillance coupled to timed-delivery that is often commenced before term. Anti-hypertensive drugs only have a limited effect, and must be used carefully because they carry the potential for reducing uteroplacental blood flow [27], [28]. Early delivery (often by Cesarean section) is advised to minimize maternal clinical complications, but may cause negative consequences to the mother in the next pregnancy [29]. While the early delivery may shorten the baby's exposure to the stressful environment in the womb [30], [31], it is associated with additional time spent in the NICU (neonatal intensive care unit), and additional costs. Also, premature babies are more susceptible to developing diseases later in life (fetal programming) [24], [29].

In recent years, the life-long complications following PE have become apparent for the mother as well. It has been shown that women’s longevity after experiencing severe PE is approximately 10 years shorter [30]. PE during pregnancy is also followed by an increased risk for developing cardiovascular diseases (CVDs) and diabetes mellitus (DM) within the next ten years [32], [33], [34], [35], [36], [37]. The newborn also experiences long term complications including obesity and diabetes already in adolescence [38], [39]. Living under stressful conditions in the womb during early onset PE influences fetal programming and shapes the newborn’s response to stress in a manner that leads to adulthood diseases [31]. Accordingly, the costs of treating the short and long-term complications of PE are cumulative [18], [24], [29].

Sadly, PE's societal challenge is increasing due to the world's growing epidemics of obesity [40], [41], [42] and DM [43], [44]. Obese women (BMI > 35) are twice as likely to develop PE during pregnancy (in central Europe PE prevalence of obese pregnant women is 5.5–7% vs. 2.5% in the entire population), and the highest frequency (20%) of occurrence is among women with DM. PE is also higher among women who conceive through assisted reproductive technologies (ART). According to the European prenatal health survey [44], there is an increasing trend for advanced maternal age in pregnancy, and the frequency of developing PE is approximately 6–8% among women above 40 years. Obese women who become pregnant at advanced maternal age have 21% risk for developing PE and later CVDs. The American Heart Association has published a white paper describing the life-long complications related to PE, and its association with a higher susceptibility to develop CVDs, and has issued specific guideline for the clinical surveillance of this group [45], [46].

Based on the above, the European Commission in its evaluation of drugs for rare diseases [47], as well as the USA Food and Drug Administration [48], have both independently decided to give the status of “orphan drugs” for drugs designed to fight PE. The underlying rationale was widely covered by Hahn [49] who also described the different causes of morbidity and mortality derived from PE.

### 1.3 Treatment of Preeclampsia

In the past decade physicians were increasingly obliged to resort to “off-label” use of drugs for evaluating their benefit to fight PE [50]. The purpose of the trials were to see if the drugs may have alternate purposes and beneficial effects to women with PE, a condition with no true medical intervention, yet. The idea behind this concept is that these drugs have already undergone safety testing and have been approved for use in pregnant women, thus reducing the cost of their evaluation in treating PE.

Aspirin is already incorporated into the guidelines of both the WHO and the UK national institute of clinical excellence (NICE) for the prevention of PE. However, according to the current guidelines, > 30% of all pregnant women should be treated. The experience has been that there was low compliance of physicians in prescribing the drugs, and of patients using the drug. Also, when aspirin is used in a large number of patients, its unfavorable side effects start to be noticed. Some of this review’s co-authors were involved in the ASPRE clinical trial for evaluating aspirin versus placebo in the prevention of PE. In the ASPRE trial, approximately 1,800 patients identified in the first trimester as being at elevated risk to develop PE based on history, biophysical and biochemical markers, were randomized to 150 mg/day aspirin vs. placebo taken at bed time from 12 to 36 weeks of gestation. The study showed an 83% reduction in the frequency of early PE, a 62% reduction in preterm PE, and a non-significant effect in the reduction of PE at term [51]. The ASPRE study reached a much higher effect compared to the average 10% reduction reported in an earlier meta-analysis [52]. The differences are attributed to the larger aspirin dose used in ASPRE (150 mg/day compared to 80, 60 or 40 mg/day in most previous studies), earlier start of the treatment (at 12 weeks compared to starting at gestational week 18, 20, 24 and later in most previous studies) as described in the meta-analysis of Roberge et al. [53].

Several studies evaluated the effect of generic drugs widely used for preventing CVDs including low molecular weight heparin [54] and sildenafil citrate as drug candidates to prevent PE [55]. Other generic drugs with a broader spectrum such as calcium supplementations have been evaluated as well [56]. The rationale for using these drugs relates to the pathophysiological features that CVDs share with PE, and the hope that the drugs will enhance vasodilation and/or provide anti-thrombotic actions [57], [58]. Calcium supplementation was proven effective mainly in adolescence pregnancy in Columbia [56]. Sildenafil incorporated in a large randomized study was found to generate no effect in PE prevention (Zarko Alfirevic, World Congress in Fetal Medicine, Ljubljana, Slovenia, 6/2017).

Metformin, usually used to fight metabolically derived DM to moderately reduce weight and blood sugar levels, was evaluated in pregnant women to assess its effect on weight gain, and frequency of gestational diabetes mellitus (GDM), PE, and other pregnancy disorders. It was discovered that metformin is very effective in preventing PE, mainly PE at term (~ 50% reduction) among obese women (BMI > 35) [59] in addition to its effects on reducing weight gain. In these studies metformin has not shown to affect the frequency of other pregnancy disorders.

A preliminary study of PE prevention was also conducted with statins, usually used to reduce blood lipids and prevent atherosclerosis. The rationale was that these drugs act on the ratio between soluble fms-like tyrosine kinase-1 (sFlt-1), an anti-angiogenic factor, and placental growth factor (PlGF), a pro-angiogenic factor. This ratio is “balanced” in normal pregnancy and increased in atherosclerosis and PE [60]. A small-scale clinical study has shown that statins are potent sFlt-1/PlGF ratio modifiers in PE [60]. It was also proven to be effective in preventing PE in women who develop a form of PE related to having the phospholipid syndrome, a condition known to be a strong risk factor for developing PE [61]. A very large study is now being conducted by the Fetal Medicine Foundation (FMF) in the UK, Spain and other European countries to explore the statins impact on term PE prevention.

Additional efforts to develop medical therapies are found at the preclinical level in *in vitro*, *ex vivo* and *in vivo* research. This includes a number of compounds unrelated to treating CVDs. Among them is Sofalcone, a drug currently tested only in tissue culture that potently activates the antioxidant nuclear factor (erythroid-derived 2)-like 2/HO-1 pathway, decreases sFlt-1 production, and ameliorates endothelial dysfunction [62].

A different mechanism involves the potential use of proton pump inhibitors (PPI). It has been found that blocking PPI leads to decreased sFlt-1 and soluble endoglin (sENG) secretion and endothelial dysfunction, dilation of blood vessels, decreased BP, and antioxidant and anti-inflammatory properties [63]. Esomeprazole, another proton pump inhibitor that is also used for gastric reflux, is also being evaluated in phase II clinical studies to treat early onset PE (PIE Trial) [64].

Treatment with recombinant human anti-thrombin versus placebo, combined with expectant management failed to show any benefit in reducing PE or increasing time to delivery, indicating that - at least when used at the third trimester of pregnancy - this treatment strategy is of no value [65].

The evaluation of generic and new drugs, as described above, has indicated both the benefits and limitations of current experimental and practice treatments. From all the studies completed to date the ASPRE study for aspirin prevention of preterm PE (PE developed before 37 weeks of gestation) and the metformin study to prevent PE in obese women showed the largest significant reduction in PE frequency in the target group following treatment.

However, even in the ASPRE and the metformin studies, the high risk group who received placebo had approximately 10 times more patients who were at high risk than the ones who actually developed the disorder (in ASPRE - 822 patients in the high risk group treated with placebo of which 82 developed PE (35 preterm PE)) [51]. These findings showed that with today's tools, based on pregnancy and medical history, serum and biophysical markers, the group identified as being at high risk includes quite many patients who will not go on to develop the disorder. Thus, it appears that not all patients in the high risk group are similar and neither aspirin or any other drug could prevent all cases in the same way. Better risk stratification methods are required to narrow down who should be treated, and how [66], [67].

The limitations of risk stratification were recently covered by the authors of the international SCOPE study [68]. These authors stated that: “the ability to predict PE in healthy nulliparous women using clinical phenotype is modest”, and indicated the need for “personalized clinical risk profiles to which biomarkers could be added”.

### 1.4 Preeclampsia – A Multi-factorial Disorders

Major leaders in the field of placental research and physiopathology listed various physiological pathways that may cause the development of PE. The International Society for the Study of Hypertension in Pregnancy (ISSHP) has issued a universal definition of PE [69], [70]. However, it is well established today that the disorder's symptoms may develop due to different underlying pathways [71], [72], [73], [74], [75], [76]. Some studies based their evaluation on the imbalance of trophoblast turnover, leading to aponecrotic release of factors into the maternal circulation [70], [71], [76]. Oxidative/nitrosative stress appears to be a major contributor [70], [71], [72]. Inflammation and modulation of the pro- and anti-angiogenic factors (such as sFlt-1, sENG, PlGF and others) have been widely proposed as the origin of the disorder [71], [73], [76]. A combined effect of oxidative stress and angiogenic imbalance was listed as core disease origins related to placental factors [71], [72], [73], [76]. Recent models have postulated that maternal cardiac insufficiency is responsible for the development of PE [74]. The development of the disorder is also attributed to immune rejection of pregnancy by the maternal decidua facing invading trophoblasts that carry paternal genes. Studies by several authors have found a correlation between a higher frequency of PE in nulliparous (compared to multiparous) women and the lack of maternal immune tolerance to the invasion of paternal genes. They also correlated the higher frequency of PE in patients with the same partner who experienced a long (> 7 years) separation between first and second pregnancy. These studies also found a higher PE frequency in pregnancies derived from donated eggs and sperm, and indicated that this may be due to immune rejection of the donor's unknown genes causing a resistance to placental trophoblasts and to a rejection of migrating trophoblasts that invade the uterine wall [77], [78], [79].

### 1.5 When to Treat? Whom to Treat?

The multifactorial origin of the disorders and the identified multiple pathways that might lead to PE development have significantly complicated efforts to develop PE prevention and treatment. In terms of when to treat, there are, in general, two major strategies: 1) use of preventive agents for treating patients at risk, and 2) treating at the time of symptoms.

The current leader in the prevention approach is low dose aspirin, as discovered in the ASPRE study [51]. Drugs such as heparin [54], sildenafil citrate [55], calcium supplementation [56], metformin [59] and statins [60] have all been evaluated in the context of preventative treatment of non-symptomatic patients selected based on various risk evaluation algorithms using history, biochemical and biophysical markers.

The other strategy focuses on treating symptomatic patients who have already developed clinical symptoms of PE. These patients are already undergoing cardiovascular stress (both the mother and her fetus). According to this strategy, the efforts are directed towards preventing disease exacerbation, at least for a short while, and thereby extending pregnancy to reduce the potential impact of prematurity, especially for early onset severe PE. Antihypertensive drugs used in a randomized trial have been shown to slow the progression of the disorder by 7–11 days, thus reducing the level of prematurity [28]. MgSO_4_, which has been proven effective in preventing stroke and convulsion in eclampsia, is also used in many places to slow the progression of severe symptoms, although there is currently no research proof for this use [80]. Anti-thrombin introduced at gestational week 24–28 was found ineffective [65]. The direction that appears to offer hope is the use of apheresis to remove excess soluble sFlt-1 from the maternal circulation in cases of very severe early onset PE [81]. Another strategy for preventing PE, mainly the type associated with growth restriction, involves adenoviral delivery of VEGF through the uterine arteries [82], [83].

The question of whom to treat applies to both prevention and treatment strategies. The prevention strategy relies on markers only (including prior maternal risk factors), and the best accuracy today is a 76% detection rate for 90% specificity, associated with treatment of many false positive cases [51]. The treatment strategy relies on both, symptoms and markers. The markers at this stage of clinical symptoms are very accurate, e.g. especially sFlt-1 or the ratio of sFlt-1/PlGF offer > 90% sensitivity and specificity. However, the treatment is implemented at a time of multiple secondary and tertiary complications, and is thus not easy to perform.

The decisions in selecting whom to treat are challenging. In the ASPRE study, the prediction algorithm has generated 93% detection rate for early PE, 76% for preterm PE, and 44% for PE at term, all at 90% specificity [51]. As a result, the high risk group for preterm PE was composed of ten times more patients than those who eventually developed the disorder. The treatment reduced preterm PE by 63%. The ASPRE prevention efficacy is the best reported today, and it meets economic cost/benefit criteria for using aspirin for PE prevention [29]. Yet, it appears that there is a need for an approach to further size down the high risk group, and to increase the likelihood of prevention among treated patients.

We therefore see the need for a personalized approach that is set forth along the following assumptions:

• PE is the common final phenotype of a multi-factorial disorder derived of various molecular pathways [69], [70], [71], [72], [73], [74], [75], [76], [77].
• Each molecular pathway leads to the development of the PE phenotype either alone or in conjunction with other pathways.
• The strategy then should be based on identifying molecular markers for particular affected pathway(s), and use a prevention/management approach that is linked to the specific impaired pathway.
• The marker(s) could then be utilized for monitoring the progress of treatment/prevention.

Accordingly, developing a targeted treatment will be based on a set or sets of molecular-based assays related to a single disease subtype derived from impaired manifestation of this molecule. A personalized approach can then target the treatment to those who can be identified according to the said assays to identify the impaired molecular marker(s) and/or specific biological and physiological functions depending on it. The hope is that based on such an individualized treatment strategy, the downstream phenotypic disease symptoms can be prevented or at least reduced [84].

## 2 Targeted Approach to Combat Preeclampsia

The genetic origin of PE has been evaluated in a variety of studies [79], [85], [86]. Previous PE of the pregnant woman and/or of her mother and sisters has already been identified as a major risk factor, along with black ethnicity [87], [88]. We use the term black ethnicity as statistics have shown higher rates of PE in Africa compared to Europe, and among Afro-Americans and Afro-Caribbeans compared to Caucasians.

Specific gene mutations have been identified in certain populations. One example is the STOX-1 mutation identified in a Dutch cohort to be associated with high risk for developing PE [89]. Mutations in complement regulatory proteins were identified as a predisposing factor for developing PE [90]. The list of genes implicated in both PE and CVDs is long and includes the p-53 pathway, inflammatory chemokines, interleukin signaling, B-cell activation, PDGF, TGF-β- and integrin signaling, Alzheimer disease pathways, apoptosis, graft-versus-host diseases, allograft rejection, steroid hormone synthesis, type I/II DM, and VEGF, GNRH or Notch signaling [91].

In this respect, it is interesting to consider the specific gene mutations that have been identified among “native” Andeans and Himalayans living at high altitude. These mutations enable them to further augment a pregnancy-associated rise in erythropoietin (Epo) for a successful vascular adaptation to pregnancy at high altitude. Epo, a pleiotropic cytokine, has important angiogenic and vasoactive properties. The native Andeans/ Himalayans who carry the mutations are capable of adapting to the increased demand for oxygen during pregnancy and have genetic advantages over newcomers to high altitude, who suffer from a very high prevalence of severe PE during pregnancy [92].

Taken together, we can see that PE is associated with a broad range of gene activation/silencing events that are implicated in the pathophysiology of the syndrome. The list is long and includes various genes. If the approach above is correct, in coming years we anticipate seeing the development of different strategies for fighting PE that are related to metabolic pathways, the immune response, inflammation, oxygen supply, cell communication, etc. Gene enrichment is thus conducted as the next generation of PE biomarkers not only to evaluate the risk for developing the disorder, but also in the context of directing treatment. The individualized medicine approach for PE aims at developing treatment directed towards a specific molecular target known to have an impaired function and/or expression/sequence in the context of elevated risk for developing the disorder in a particular patient. For example, patients carrying mutations/polymorphisms in these novel biomarkers will be included in clinical studies directed for treating the disease through an individualized pathway that is specific to the respective impaired gene.

While PE is a multi-factorial disorder, the assumption is that one can identify single gene impairment and its polymorphic variants, and develop exclusive treatment that focuses only on this molecule among the affected population. Treatment strategies will be based on understanding the signaling pathways associated with this molecule, and will aim to improve the functionality of this molecule, either by augmenting its expression, replenishing the “correct” molecular variant, replacing the defective one, or recruiting other means to bypass the impaired pathway or interfere with its potential harmful impact.

Clearly, multiple damaged molecules may be identified to be co-expressed and may underline the more severe disease phenotype, thereby creating pathways based on two or more molecules as the next steps.

### 2.1 This new Approach Would be Specific and Individualized

It requires the use of a battery of tools, and not a single test. Specific assays need to be introduced for identifying patients with impaired specific gene targets either in its primary sequence, RNA or protein expression, or other targets and methods. In turn, identified subjects will be stratified for inclusion in clinical studies in the context of a specific molecular pathway that is implicated in the development of PE. Preclinical studies would then be directed to link between biomarkers and model systems to assess the degree of their impairment and the benefits of correction. Treatment strategies may then be introduced for replenishing with the proper protein (or a fraction of it), or, where appropriate – providing its agonist or antagonist, or similar methods [93], [94].

In conclusion:

• PE is a multi-factorial syndrome.
• The new approach for fighting PE focuses on individualized medicine, aiming at developing treatment with a specific biological target/molecular entity to identify those who suffer from a specific subtype of PE that is linked to a specific molecular pathway.
• The affected pathway could be associated with impaired placental development, impaired RNA/protein expression, DNA mutations, etc.
• This way, the multifactorial origin of PE can be “dissected” from a “collection of diseases”, which merge during pregnancy into a common final symptomatic phenotype, with well-defined signaling pathways that all add to the disease phenotype. The severity may be related to one particular pathway or to pathway combinations.
• This approach may sound difficult, but offers hope, as it will enable directing treatment to the origin of the disease and not to its phenotypic symptoms, and will treat only the target group, and is likely to attain better outcome.
• If successful, the approach can subsequently be adopted to develop strategies for combating a broader spectrum of PE sub-types related to various underlying signaling pathways and relevant molecules.
• After a successful implementation of this approach for fighting PE, any other multi-factorial syndrome can be approached in a similar manner.

Our first test case to this approach is placental protein 13 (PP13).

## 3 The case for Placental Protein 13 (PP13)

The research for drug targeting to PE with placental protein 13 (PP13) started in 2013 [94]. PP13 is a relatively understudied molecule among the PE biomarkers compared with the angiogenic/anti-angiogenic factors (e.g. sFlt-1, endoglin), but it has attracted interest due to several unique features.

**Fig. 1:**
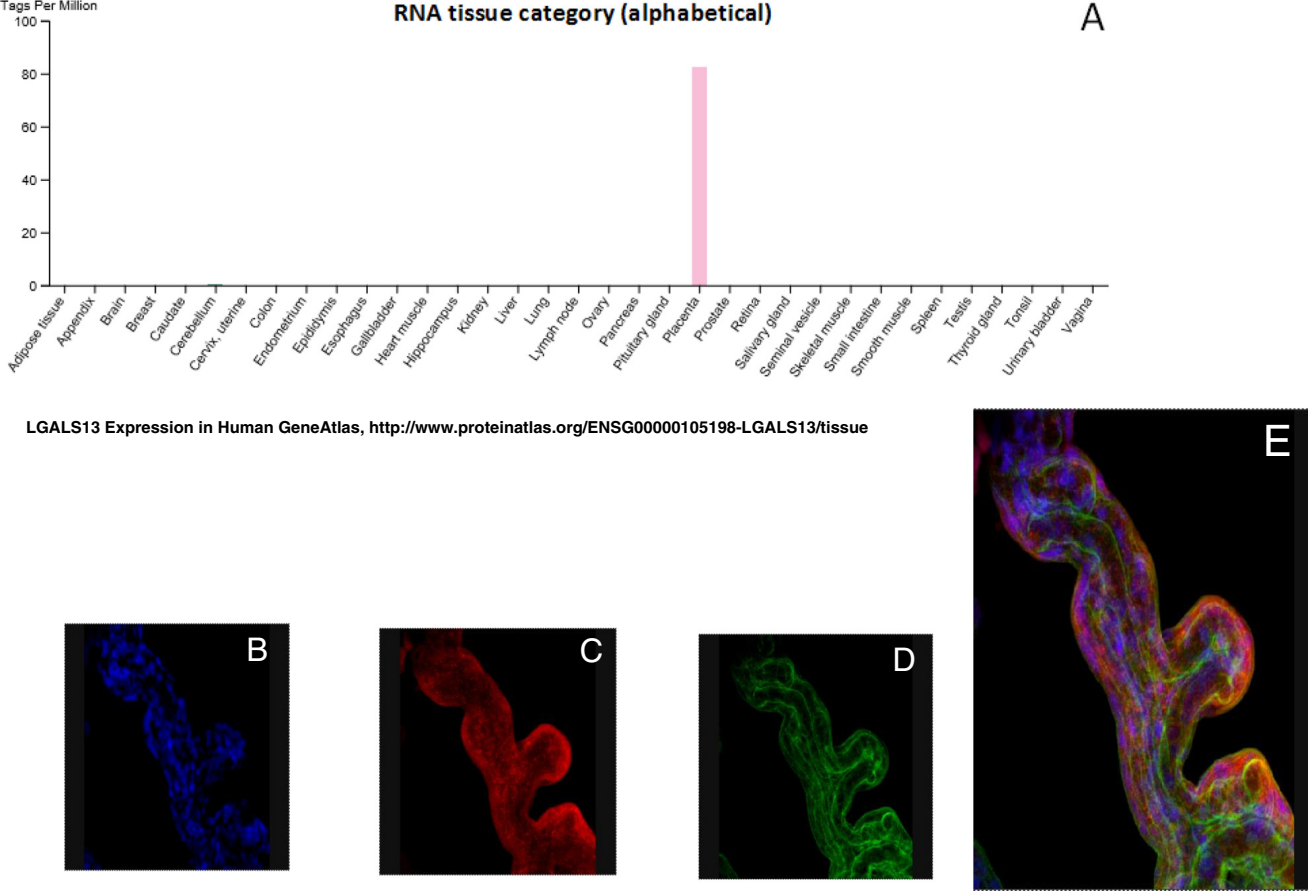
PP13 expression (A) Exclusive expression of PP13 mRNA in the placenta compared to other human tissues according to the Human GeneAtlas. (B)–(E): Co-Localization of PP13 on a syncytiotrophoblast villous with actin compared to the nuclei. (B) Stain with DAPI (blue) for the nuclei, (C) Stain with TRITC conjugated anti PP13 antibodies (red), and (D) Stain with FITC conjugated anti actin antibodies (green). (E) A tri-dimensional image of the co-localization of all three together.

The molecule was discovered in the 1980's and its sequence was published in 1999 [95]. PP13 is a placenta-specific molecule (Fig. 1A). In adult tissues its expression is limited to the placenta. It has also been identified in rare fetal tumors [95]. This protein is a galectin with a high affinity for sugar residues of other proteins [96], [97], and is encoded by the LGALS13 gene located on chromosome 19q13 near several other galectin genes [95], [96], [97], [98], [99]. In this context, it is worth citing the work of Freitag et al. [100] who have shown that interfering with angiogenesis mediated by another galectin - Gal-1 – contributes to the pathogenesis of PE.

PP13 is mostly expressed in the villous syncytiotrophoblast of the placenta [101], [102], and its expression has been determined in both primary cultured placental cells, placental villous explants, and in placenta-derived cell lines (e.g. BeWo cells), especially when they are stimulated by forskolin to undergo fusion and form syncytia [103]. PP13 localization in adult tissues appears to be mainly related to the placental villi (Fig. 1B) [97], [98], [102], while PP13 is also found in the placental bed and the fetal endothelium [97], [98], [102], [104], [105].

Cross linkage analyses have shown that PP13 has a high affinity for sugar residues of annexin IIA and beta & gamma actin [97]. The 3D conformational changes of PP13 in the presence of sugar residues have been analyzed, and the amino acids involved in sugar binding were identified [106], [107] along with an analysis of these amino acids during evolution [95], [96], [97], [98]. Using multiple labels of villi with anti- PP13 and anti-actin or anti-annexin 2 antibodies, and with nuclei stain with DAPI (Fig. 1B-E), they appear to have close proximity along the syncytiotrophoblast. PP13 also displays high affinity for the beta galactoside of the blood group B antigen [108].

Circulating PP13 has been detected in the maternal blood from the 5th week of gestation [101], and its level slowly increases during the course of pregnancy [102], [104], [109]. Cultured isolated placental villi were shown to release PP13, a process that was augmented after adding aspirin to the culture medium [110]. Studies have also shown increased PP13 expression induced by aspirin [111]. In this model, transfection of the placenta-derived BeWo cell line with the PP13 construct was followed by high PP13 expression that was increased by forskolin-induced differentiation, and by exposure to a calcium ionophore [112].

PP13 as a biochemical serum marker of PE in pregnant women has been reported in more than 20 studies [96], [108], [109], [110], [114], [115], [116], [117], [118] using various assay platforms, different cohorts of pregnant women, and multiple prior risk populations derived from different ethnic and geographic origins [94], [113]. According to these studies, PP13 as a single biomarker has an 83% detection rate for 10% false positive rate for early PE (< 34 weeks), 66% for preterm PE (< 37 weeks) and 47% for all cases of PE combined [69]. Together with measurement of the Doppler pulsatility index of the blood flow through maternal uterine arteries or additional markers, the prediction of PE in the first trimester was 93% for preterm PE combined with fetal growth restriction [117] and higher still when PP13 is further adjusted to the blood group [108].

Longitudinally, the PP13 level increases modestly from the first to the third trimester. This slow increase is a combined effect of increased numbers of placental villi during pregnancy (larger surface area), while the level released by each single villus is actually decreased, which is compensated for by the increased placental surface area. However, the level of increase is sharply augmented in women entering the symptomatic stage of the disorder along, presumably due to an increased release from individual villi [102], [104], [105], [108], [109], [110]. PP13 disappears from the maternal circulation within 2 to 5 weeks after delivery [94], [102], [109].

PP13 mRNA was identified in the placenta by hybridization studies [99]. At delivery, placental PP13 RNA in PE cases is significantly lower compared to normal control [104], [105]. There are a number of studies that have shown that the level of PP13 mRNA in the maternal circulation is lower in PE cases compared to controls, and that the lower level can be detected throughout pregnancy (first and third trimester) in either the maternal circulation or the placenta [105], [119], [120], [121]. As the level of circulating free PP13 mRNA in maternal blood is very low [119], [120], [121], and its half-life very short, PP13 mRNA tests available today offer a low detection rate, and message may not be a good marker on its own. However, when coupled with other prior risk factors (previous PE, ethnic origin, BMI and advanced maternal age), the detection rate of PP13 mRNA test becomes higher [121].

**Fig. 2:**
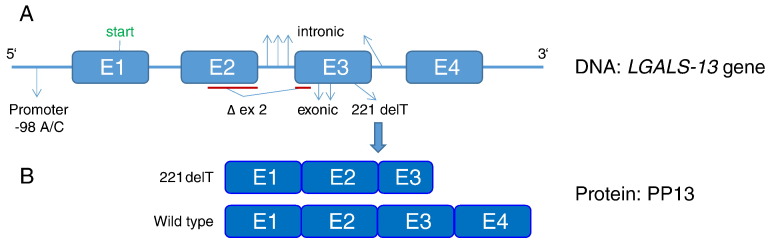
PP13 (LGALS13) DNA and protein (A) The configuration of LGALS13 made of 4 exons (E1-E4). The 3′ and 5′ ends are shown. Intronic and exonic mutations are marked. Note the − 98 promoter variants (with A and C nucleotides) and the thymidine deletion in position 221 (221 delT) of the open reading frame. (B) Exon construction of the wild type PP13 protein and the truncated variant (221 delT) protein that was used for expression experiments.

PP13 polymorphisms. As mentioned before, PP13 has been sequenced, cloned, expressed, and purified. Its DNA configuration presented in Fig. 2, indicates that it has 4 exons (E1–E4, Fig. 2A). The molecular weight is 16 kDa for the monomer although it is more stable as a dimer, and the polypeptide has 0.6% carbohydrate and 0.8% lipid co bounds to it [95], [96], [97]. The discovery of polymorphic PP13 variants [121] has driven research to identify the potential correlation between reduced PP13 mRNA in PE [119], [120], [121] and the presence of PP13 polymorphic variants [122]. Most variants are rare and their very low frequency is a challenge for reaching statistical significance in molecular investigation. However, two variants show sufficient prevalence to drive research on their potential role as molecular markers of PE.

(1) The promoter variant - The -98A/C promoter variant (Fig. 2A) is identified with an “A/A” genotype (homozygous to the adenosine nucleotide) or “C/C” genotype (homozygous to thecytosine nucleotide) or an “A/C” genotype (heterozygous form). In first trimester blood samples from pregnant women who subsequently had a healthy delivery without PE, all three genotypes are in Hardy Weinberg equilibrium. The level of A/A genotype is always the largest (~ 65%), but in pregnant women who subsequently develop PE, the level of A/A is significantly higher (~ 82%) and the three genotypes are no longer in Hardy Weinberg equilibrium (Table 1). Accordingly, it appears that having at least one “C” allele (e.g., not only the C/C but also the A/C genotype) protects against the development of PE [123], [124]. Similar results have been obtained in a South African Cohort as well as in a London cohort [123], [124]. Carriers of the A/A variant had an adjusted Odds Ratio of 2.45 [1.16–5.20] for developing PE, while the carriers of the C/C and the A/C genotypes are protected from developing PE.,The presence of the A/A genotype in association with obesity (BMI > 35), history of previous PE, African ethnicity and pregnancy at advanced (> 40) maternal age, is accurately predicting a very high risk for developing the disorder with adjusted Odds Ratio of 15.6 for term PE and 11 for all PE cases [124]. Obesity was the major confounding variable, indicating the relevance of improving the selection of obese women for targeted prevention.

**Table 1:** The frequency of promoter variants The − 98 promoter genotypes were determined by DNA extraction followed by DNA amplification by PCR and sequencing of circulating free DNA in maternal plasma of pregnant women in the first trimester revealing the A/A, A/C and C/C genotypes in the control and various PE sub-types. Modified from Madar Shapiro et al., Ref. [124].

	Unaffected control (n = 196)		All PE (n = 67)		Preterm PE (PE 34^0^–36^6^) (n = 18)		Term PE (PE > 37 w) (n = 49)	
Genotype	N	%	N	%	N	%	N	%
A/A	132	67.3	55	82.1	13	72.2	42	85.5
A/C	48	24.5	9	13.4	3	16.7	6	12.2
C/C	16	8.2	3	4.5	2	11.1	1	2.0
*p*			*p* = 0.068		*p* = 0.730		*p* = 0.032
  It has been shown that binding of the TFAP2A promoter activator is three times higher to “C” in the -98 position than to “A” in this position, indicating that the presence of “C” is anticipated to induce higher PP13 expression. PP13 promoter reporter expression studies have been conducted after transfecting BeWo cells with PP13 having “A” or “C” in the − 98 promoter region using Luciferase assays. In non-differentiating BeWo cells, a higher PP13 expression with “− 98C” was measured compared to “− 98A” (p = 0.04). Forskolin-induced differentiation of BeWo cells led to a 4.55 fold increase in PP13 expression with the “− 98C” clone compared to a 3.85 fold increase with the “− 98A” clone (p < 0.001) [124]. Altogether, it appears that the higher risk for PE is associated with the “A/A” genotype may be due to reduced PP13 expression among obese women who had PE in previous pregnancies and are of African ethnicity. Screening to identify this sub group PP13-related high risk for PE can be performed by real time PCR.
(2) “Truncated” – A thymidine deletion in position 221 of the open reading frame of PP13 (located on the region encoding for exon 3) (Fig. 2A) was discovered by Gebhardt et al. [122] in a black and colored pregnant women cohort in South Africa. The mutation is accompanied by a shift in the open reading frame, and the generation of an earlier stop codon coupled to the expression of a shorter PP13 variant (“truncated” or “delT_221_”) lacking exon 4 and a fraction of exon 3 [122] (Fig. 2B). Heterozygous pregnancies of this mutation develop early severe PE and have very low level of PP13 due to an impaired PP13 molecule. The truncated PP13 variant is rare and accounts for about 7% of the cases of severe early PE (5 in 10,000 pregnancies). However, in South Africa, where PE has a high prevalence, carriers of the heterozygous mutation are close to 1:3,000 of the pregnant population, and they have an 89% positive predictive value for developing PE. Carriers of the homozygous form of DelT_221_ mutation are non-viable and are associated with early pregnancy loss [122], indicating that there is a need for at least one copy of the wild type (w/t) PP13 for a successful pregnancy.
  Truncated and w/t PP13 were expressed in bacterial (*E. Coli*), and the polypeptides were purified [124]. When added to leukocytes derived of the maternal decidua, w/t PP13 but not the truncated variant caused leukocyte apoptosis [98], [125], [126]. It was postulated that w/t PP13 plays a role in rendering the mother immune-tolerant to pregnancy [98], [125], [126] while the truncated (DelT_221_) variant fails to do so. This can be attributed to the loss of carbohydrate binding capacity due to the absence of two amino-acids involved in the carbohydrate binding domain, and of two additional amino acids supporting carbohydrate binding [106], [107]. The immune tolerance provided by PP13 is thus estimated to be related its carbohydrate binding capacity [98], [125], [126].

Modified from Huppertz et al., Ref. [94], and from Sammar et al., Ref. [125].

Taken together, it appears that polymorphic variants of the PP13 molecule may serve as molecular markers to determine impaired DNA/RNA structure/expression using immune-assays and PCR tools. Patients identified positive in these assays represent a high risk group for evaluating PP13 as a candidate for targeted therapies to fight PE.

### 3.1 PP13 replenishing studies

**Fig. 3:**
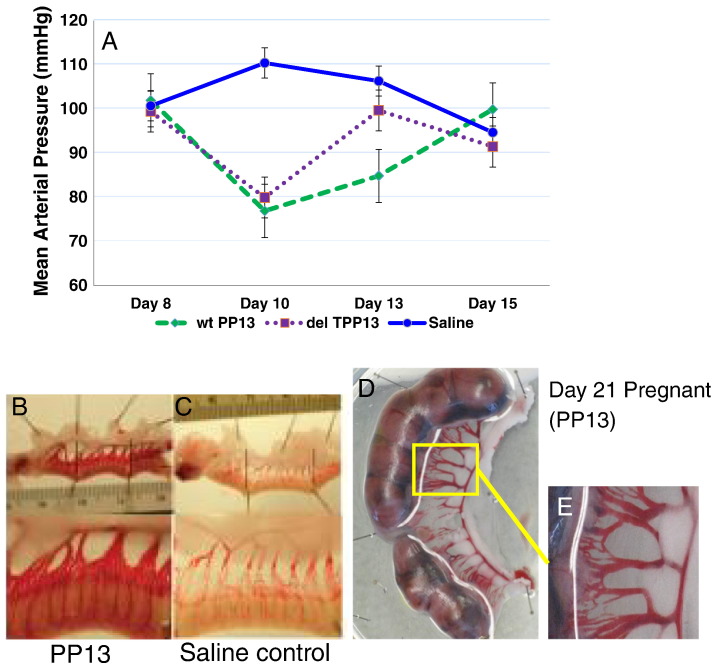
Blood pressure changes induced by PP13 A – Changes in mean arterial pressure in pregnant rats were induced by using peristaltic pumps implanted inter-peritoneally on day 8 of pregnancy for releasing their content until day15. The three animal groups had pumps that released w/t (n = 9) or truncated PP13 (n = 6) or saline (n = 6). (A) Release of w/t and truncated PP13 resulted in hypotension throughout the duration of PP13 release from the pumps. Modified from Gizurarson et al., Ref. [128]. (B–C) Increased diameters of the uterine vasculature with PP13 (C) compared to saline control (B) as detected in non-pregnant rats. Animals were subjected to continuous release from implanted pumps that released their content for seven days; the rats were sacrificed six days later (13 days overall), on gestational day 21. (D) Increased diameters of the utero-placental vasculature in pregnant rats exposed to PP13 release from intra-peritoneal 7-day release mini-osmotic pumps implanted on day 8 of pregnancy. All animals were sacrificed on gestational day 21. Inset: higher magnification of a given section from the pregnant rat exposed to PP13.
• The w/t PP13, but not the truncated variant, also induced significant utero-placental vessel dilation and/or growth (Fig. 3B–E). This was identified in both non-pregnant (Fig. 3B&D) and pregnant rats (Fig. 3E) [125], [128]. Arteries were expanded by about 45%, while veins were expanded by about 50% (for veins see Table 2) [125], [128].

PE is a primarily a human hypertensive pregnancy disorder, while gestational hypertension is a pregnancy disorder identified across the mammalian kingdom in many of the small and large mammalians. Many also develop kidney problems and suffer from elevated urine protein during pregnancy [133]. The “full blown” form of PE is, however, limited to primates and is associated with the evolution of deep placentation [126]. This makes studies in animals “tricky” for extrapolation to the effect on primate and human pregnancy disorders. However, with an awareness of these limitations, and knowing that the phylogenetic expression of PP13 is limited to pregnant primates [99], an extensive set of preclinical studies was conducted in gravid rodents to assess the impact of PP13 in pregnancy:

• Initially, a single PP13 dosage injected intravenously into gravid rats and rabbits during the second trimester of their pregnancy has resulted in a reversible ~ 30% reduction in blood pressure [94], [127].
• In a second set of experiments, peristaltic pumps were implanted into gravid rats to enable continuous release of PP13 initially in the third trimester of pregnancy, and in a third set of experiments, at the beginning of the second trimester of pregnancy [127], [128]. Applied from day 8 of pregnancy, the pumps released PP13 until day 15 accompanied by a reduction of the blood pressure that reached the lowest blood pressure at 10 days of pregnancy (two days after the start of the release), and then slowly returned to normal by pregnancy day 15 (compared to saline control) (Fig. 3A). Hypotension was recorded for both w/t PP13 and the truncated PP13 variant, although the effect of the latter returned to normal in a faster pace (Fig. 3A) [128].

**Table 2:** PP13 induced increase of venous diameters Changes in venous diameters in gravid rats at day 21 of pregnancy following previous exposure to slow release of w/t (n = 9) and truncated PP13 (n = 6) compared to saline control (n = 6). The pumps were implanted in pregnancy days 8, and PP13 was detected in the animal blood until day 15. Note that, the closer the vein is to the placenta, the larger the effect. Only w/t PP13 was able to induce enlargement of venous diameters, while the truncated form (delT PP13) was unable to do so. Bolded (w/t PP13) values are significantly different from controls (*p* < 0.001). Modified from Gizurarson et al., Ref. [128].

Type of vein	Control (n = 6)	PP13 (n = 9)	delT PP13 (n = 6)
	Mean ± SD	Mean ± SD	Mean ± SD
Post-placental vein	0.28 ± 0.07	**0.41** ± **0.11**	0.24 ± 0.09
Post-myometrial vein	0.55 ± 0.34	**0.61** ± **0.14**	0.33 ± 0.16
Radial vein	0.75 ± 0.32	0.91 ± 0.32	0.52 ± 0.15
• At delivery on day 21, 6 days after the blood pressure returned to normal and the slow release of PP13 was ended, a significant increase in placental (46%) and pup (10%) weights was detected in the w/t PP13 group, whereas placental and pup weights were significantly decreased in the truncated group (Table 3) [125], [128]. Pup numbers, however, were unaffected.

**Table 3:** Assessment of placental and pup weights after long exposure to w/t and truncated PP13 PP13 was slowly released from peristaltic pumps implanted inter-peritoneally in gravid rats on day 8 of pregnancy, and released saline control or w/t or truncated PP13 until day 15 of pregnancy. Animals were sacrificed on day 21 followed by weighing the placenta and the pups. Bolded values are significantly different from controls (p < 0.01). Modified from Gizurarson et al., Ref. [128].

Parameter	Control (n = 6)	PP13 (n = 9)	DelT PP13 (n = 6)
Pup weight (g)	2.24 ± 0.33	**2.46** ± **0.35**	**0.93** ± **0.11**
Placental weight (g)	0.45 ± 0.07	**0.64** ± **0.11**	**0.37** ± **0.09**
Number of pups (n)	12.5 ± 4.9	14.0 ± 1.4	13.5 ± 0.7

Table 4 summarizes the differences between w/t and truncated PP13. Both induced hypotension; however, only the w/t stimulated arterial and venous expansion along with a larger placenta and bigger pups [94], [128] indicating the importance of PP13 for avoiding growth restriction (IUGR) in addition to its hypotensive and uteroplacental vascular effects.

**Table 4:** Comparative differences between Del T and w/t PP13 variants Comparative differences extracted from the measured functional differences between w/t PP13 and its truncated variant. The analysis is based on references [94], [98], [125], [126], [127], [128].

	Systolic blood pressure	Diastolic blood pressure	Mean arterial pressure	Arterial diameter	Venous diameter	Placental weight	Pup weight
Control	~	~	~	~	~	~	~
W/T PP13	↓	↓	↓	↑	↑	↑	↑
DelT PP13	↓	↓	↓	~	~	↓	↓

### 3.2 Signaling Pathways

To identify the signaling pathways underlying these changes, isolated uterine and mesenteric arteries from both mid-pregnant and non-pregnant rats were used. After isolation the arteries were placed into arteriographs to visualize their diameters and measure lumen diameters measured in response to drug superfusion [129].

**Fig. 4:**
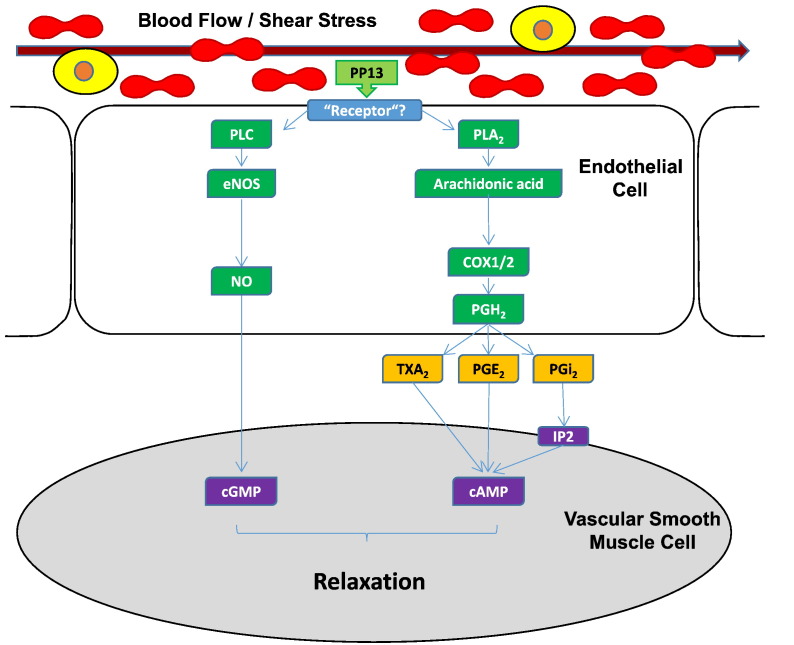
Signaling pathways affected by PP13 A scheme of the endothelium-dependent vascular relaxation pathways developed according to Drobnjak et al., Ref. [129]. Interaction of PP13 with the endothelial layer results in activation of nitric oxide (NO) production via endothelial nitric oxide synthase (eNOS), as well as metabolism of arachidonic acid (AA) to prostaglandins (PG) via cyclooxygenase (COX1/2) enzymes. NO and PG normally elicit relaxation of vascular smooth muscle cells through cGMP and cAMP, respectively. The endothelial element activated by PP13 is yet unknown. Note that, in the context of blood vessel relaxation, PP13 does not alter endothelial cytosolic Ca^2 +^ levels, and that the IP (prostacyclin) receptor is not involved, suggesting that another prostaglandin, e.g. prostaglandin E2 (PGE2) may be responsible. cAMP (cyclic adenosine monophosphate), cGMP (cyclic guanosine monophosphate), PLA_2_ (phospholipase A2), PLC (phospholipase C), PGE2 (prostaglandin E2), PGH2 (prostaglandin H2), PGi2 (prostacyclin 2), TXA2 (thromboxane 2). Scheme adapted from Drobnjak et al., Ref. [129].

• Both the uterine and the mesenteric arteries were dilated in a dose dependent manner by increasing concentrations of PP13 with a mean efficacy of 38–50% [129].
• Half-maximal vasodilation of isolated arteries (EC_50_) was achieved at 1 pM PP13, a concentration that corresponds to first trimester maternal blood levels of pregnant women [102].
• The effect was endothelial layer mediated, since vessels without the endothelial layer [130], [131] lost the ability to dilate in response to PP13 [129].
• Pharmacological analysis of the signaling pathways revealed that the effect was mediated through eNOS and prostaglandin 2 receptors (Fig. 4).

Hence, PP13 pre-conditions the utero-placental vasculature pregnancy by causing arterial expansion mediated via the endothelial layer, through vaso-dilatory eNOS and prostaglandin pathways (Fig. 4). This would likely improve blood flow and nutrient supply to the placenta and resulted in placental enlargement and increased pup weights. It is important to mention that PP13 has no typical “receptor” in conventional pharmacological terms [94], [95], [96], [97]. Therefore, it is anticipated that this effects are mediated through the sugar residues of the endothelial layer-related molecules of the eNOS and prostaglandin pathways. In this respect it is interesting to mention that PP13 has a mild phospholipase effect, and was shown to cause slow release of prostacyclin and arachidonic acid [132].

## 4 Animal Models to Evaluate PP13 for Fighting PE

The molecular markers and assays described above, and the animal studies place PP13 as a candidate for fighting PE. This should be further examined in animal models to enable the first human studies. One has to be cautious since the full spectrum of the human syndrome is not present in animals that lack the specific morphology of the human placenta. However, each of the PE animal models published so far replicates certain elements of the PE pathogenesis (e.g., hypertension and proteinuria, endothelial dysfunction, impairment of the nitric oxide system, over-activation of the systemic inflammatory response, elevation of circulating proteins that interfere with angiogenesis, etc.) [133], [134], [135]. Here we focus on a few animal models in an attempt to analyse how they can be utilized.

### 4.1 The Reduced Uterine Perfusion Pressure (RUPP) Model

The RUPP model involves clamping of the abdominal aorta and of the uterine arteries to induce an acute reduction in utero-placental perfusion pressure by approximately 50% to generate hypertension and proteinuria [136], [137], [138], [139], [140], [141]. The model is usually accompanied by endothelial dysfunction and IUGR with increased thromboxane sensitivity, and is independent of the renin-angiotensin system [142], [143], [144], [145].

In the context of PP13 subsequent supplementation with PP13 is anticipated to reduce BP, proteinuria and growth restriction. In this context, single horn gravid rats [136] could be used to study the impact of angiogenesis and hypertension by providing a control for implantation and placentation [81].

### 4.2 Chronic Inhibition of Nitric Oxide (NO) and the STOX-1 Model

Chronic inhibition/deficiency of the nitric oxide (NO) pathway, especially the endothelial pathway (eNOS) in pregnancy leads to certain manifestations resembling PE [146], [147]. NO is tonically controlled and locally produced by endothelial NO synthase (eNOS). Chronic inhibition of eNOS leads to a dose-dependent sustained hypertension and proteinuria in gravid animals [148], [149]. It also increases maternal and fetal morbidity and mortality in a pattern that resembles PE [150], [151]. A reduced nitric oxide signaling has often been noted in human PE as it has been recently reviewed by Osol et al. [152].

Accordingly, in a model of a sustained block of eNOS [149] it is anticipated that PP13 injection may partially reverse the effects of reduced NO signaling and, at least in part, hypertension and reduced uteroplacental perfusion [129].

Transgenic mice could be further utilized to explore this model. PP13 is not expressed in mice [97]. PP13 expression in mice with a sustained eNOS blockade should restore normal BP alone or combined with removal of proteinuria.

#### 4.2.1 The STOX-1 Model

The STOX1 Y153H mutation was initially identified as a susceptibility marker of PE in a Dutch PE cohort of sisters who had mothers that have also had PE [89], [153]. While other cohorts have shown no differences in PE frequency between STOX1 positive and negative groups, these cohorts lack the familial structure and linkage as the ones in which the mutation was discovered [153], [154], [155]. Doridot et al. [156] have shown the physiological role of STOX1 in the development of PE. Their transgenic mice model significantly overexpresses STOX1, particularly in the placenta. When the w/t females have been crossed with STOX1 homozygous knock-ins, they exhibited significantly elevated (~ 80 mm Hg) systolic arterial BP by mid-pregnancy that disappeared after delivery. W/T crosses with STOX1 overexpressing mice exhibited a significant proteinuria, due to renal capillary swelling and fibrin deposition that affects renal hemodynamics and indicates renal injury. Administration of low-dose aspirin to the STOX1-crossed mothers was accompanied by a significant attenuation of both maternal hypertension and renal fibrin deposition. Aspirin also normalized the significant decrease in litter size. In addition, these mice show increased levels of sFlt-1 and sENG in the maternal circulation [156], [157], indicating that STOX1 overexpression is associated with genes of angiogenic proteins acting in synergy with STOX1. Relatively minor alterations in placental morphology were reported indicating that ischemia is unlikely to be a direct effect of STOX1 mutation [158]. Targeted therapy of STOX1 carriers aims to cure oxidative stress (both in vitro and in vivo) via influencing gene control over mitochondrial function as it has been recently described by the Vaiman's group [159]. Accordingly, co-expression of PP13 in STOX1 mice could act to expand arteries and counteract oxidative stress.

### 4.3 The sFlt-1 Model

We have already mentioned that an imbalance of pro-angiogenic and anti-angiogenic factors appears to be a good late pregnancy indication of a widespread vascular dysfunction characteristic of early-onset PE [82], [83], [93]. Alternative mRNA splicing of the VEGF receptor molecule (VEGF-R) yields a circulating spliced variant (sFlt-1) of the VEGFR1 receptor [91]. This process was initially discovered in tumor development and was subsequently identified in pregnant women who develop PE. The circulating variant reduces the tissue availability of VEGF, and thereby inhibits its ability to support and stimulate angiogenesis [160], [161]. There are more than 200 publications on sFlt-1 elevation and reduced free VEGF and PlGF levels in PE (e.g. [82], [83], [91]).

Injection of adenoviral vector expressing the full length human placental sFlt-1-e 15a isoform induces a distinct maternal phenotype of PE in the mouse model of PE [162], [163]. PP13 co-expression can be tested in reversing the effect in a partial or complete manner [164].

In the context of PP13, co- transfection with the placental sFlt-1-e 15a isoform [162], [163], PP13 could act to prevent the development of this phenotype of PE.

## 5 Conclusions

○ PE remains a major pregnancy disorder associated with severe morbidity and mortality, and is associated with lifelong deterioration of health of the mother and her newborn [164]. So far, issues related to threats from teratogenic or future fertility complications have limited the development of new drugs to fight PE. As a result, there is a huge discrepancy between the urgent need to fight the disorder and the availability of public and private resources to finance drug development against PE. This situation has driven the regulatory authorities to assign a status of orphan drugs to drugs aiming to eradicate PE. The definition was achieved by defining PE as a unique hypertensive disorder developing only during pregnancy. It opens a new window of opportunities in the battle against PE [47], [48].
○ Many generic drugs are tested today aiming to repurpose their use in order to prevent PE, and to leverage their safe use in pregnancy to shorten the clinical validation process of using them to fight PE. This approach is strengthened by several achievements such as the ASPRE study success for preventing preterm PE with aspirin [51] and the use of metformin to prevent PE in obese women [59]. Other studies are testing statins, low molecular weight heparin, and sildenafil citrate, among others [53], [54], [55], [56]. The approach involves treating about ten times more women identified as being at risk compared to the ones who actually develop the disorder, and none have efficacies above 60%.
○ New drug strategies focus on one or two signalling pathways that lead to the development of a PE phenotype [165] to score a better success rate. Our approach focuses on PP13 as a unique molecule affecting such signalling pathways and its polymorphisms. We have developed PCR and others tests to identify patients at high risk for developing PE according to impaired structure/function of PP13 DNA or RNA expression, and PP13 features as a galectin involved in immune tolerance, and in preconditioning the uterine vasculature to the increased physiological burden of pregnancy. By having suitable ELISA, PCR, and other diagnostics, this approach enables to select high risk patients as candidates for future clinical studies.
○ A test of the -98 A/C polymorphism can identify patients with lower expression of PP13 [124], mainly among obese women. A shortage of PP13 may lead to narrower uteroplacental arteries and veins, thus leading to hypertension, smaller placentas and smaller pups [127], [128]. With a cell-free DNA, test we have been able to identify patients with the truncated variant which, in turn, is associated with impaired leukocyte apoptosis, turning the maternal tissue immune reactive to trophoblast invasion and blood vessel remodelling [98], [125], [126].
○ Animal studies revealed the effect of supplementing with the normal PP13 molecule to reduce BP [126], [127]. Long term exposure to PP13 molecules expands the effect from hypotension and vasodilation into a structural expansion of the vasculature (remodelling of the arteries and veins) that preconditions the mother for adapting to the increased cardiovascular burden of pregnancy [124], [126]. Signalling endothelial pathways for mediating the PP13 effects involve eNOS and prostaglandins [128].
○ Drug development for fighting PE is facing a very difficult impediment in entering into preclinical studies. PE development is associated with the deep placentation into the uterine wall, which challenge the immune system. Many species in the animal kingdom, especially mammals, can be induced to develop hypertensive disorders in pregnancy, and some also develop proteinuria along or in conjunction with hypertension. Yet, none of the PE models in animals can accurately mimic the full spectrum of PE in the human [97], [126]. The structure of the placenta is very different in these animals and the extrapolation to the human pregnancy model has to be undertaken with caution. Yet, animal models are valuable in guiding subsequent studies in humans, human placenta tissues, human placental cells, and placental like cell lines [101], [102], [110], [111], [112], [124]. Animal models also enable us to get a first estimate of drug potential with respect to some aspects of PE, and allow the introduction of human genes for evaluating their impact prior to testing in higher primates and in human.
○ Transgenic animals and other methods of human gene transfection into smaller and larger animal models [158], [162], [163] along with non-invasive sonographic and other imaging tools may assist in measuring blood flow and arterial structure [158], [159].
○ We foresee the role of *in vitro* models of placental cells and cell lines [94], [102], [110], [111], [112], [124] in drug development to assess immune tolerance and determine long-term structural changes in arterial diameters, angiogenesis, among others [49], [107], [166]. The course of the development includes vascular pharmacology with special emphasis on the uterine vasculature and signaling pathways for cell growth, apoptosis and angiogenesis studies [129], [145].
○ All drug development programs should be accompanied by toxicology studies to evaluate safety according to ICH/S3 (toxico-kinetics), ICH/S1 (carcinogenicity), ICH/S2 (geno-toxicity), ICH/S4 (long term use of PP13), ICH/S8 (immuno-toxicology) and fetal toxicology (no guidelines available). Controlled *in vivo* release of any drug should be followed by thorough monitoring of physiological parameters. A dose response (pharmaco-dynamics) should characterize the response together with interspecies pharmaco-kinetics (Vd, k12, k21, ke, etc.) to achieve a therapeutic index (TI = TD50/ED50), as well as MEC (minimal effective concentration), MSC (maximal safe concentration) based on different pharmacological effects that should be studied together with testing of different nano-formulations with various targeting capabilities.
○ The summary of all studies analyzing the impact of polymorphic variants of PP13 is opening a new road to the prediction the risk to develop PE and also for monitoring it during the progression of the pregnancy. Genetic polymorphism may be used for predisposing pregnant women to the risk to develop PE. Polymorphism is another reason why a unique drug will probably not be suitable to fighting all PE cases, even for a given phenotype, as it may pre-dispose patients to resistance to certain drugs. This is most probably the case for genes encoding detoxification enzymes acting in the case of aspirin to create aspirin resistance [167]. An additional example is the polymorphism of the metformin transporter that prohibits the availability of the drug to act on the liver and the intestines, thus creating resistance to metformin [168]. This issue has to be considered in any drug development program and is quite important for personalized medicine, as emphasized in this article.

Altogether, our initiative aims at bringing the PP13 research to a level that allows clinical trials in pregnant women with patients identified by diagnostic tools as the appropriate target group according to DNA mutation (DelT_221_) or impaired expression of the − 98A/A genotype. Combined with the determination of low PP13 mRNA in maternal blood and low blood PP13 protein, a battery of tools opens the road for implementing this approach. Treatment doses will be selected according to efficacy/toxicity ratios and ranges. Studies will be open- label, controlled by ethical approval and written informed consents, and conducted according to all GCP requirements. If proven effective, this strategy could lead to valorization among the respective target groups.

It is generally accepted that the development of novel therapies to treat hypertension in pregnancy is hampered by the relatively small targeted population that challenges the potential return on investment [49], [63]. Verifying a novel drug-target composite through toxicology and physiological/molecular and morphological testing in gravid animals and in human is a feasible approach based on the strong scientific base in research animals and isolated human vessels, and subsequently in human volunteers.

The market clearly signals its need for a paradigm shift in the management of hypertensive disorders in pregnancy, linking marker discovery, risk stratification and replenishing of the missed/impaired protein to create a strong biological basis for such a therapy [165].

Our initiative identifies PP13 as a candidate to be taken into the clinical arena by creating a clear pathway from new drug targets and design, into treatment and a clear guidance of identifying the respective patients and their subsequent path for treatment. In due course we may be able to evaluate the usefulness of the CRISPR/Cas 9 approach for editing the LGALS/PP13 gene, but this day is still ahead of us [169], [170].

## Funding

This study was mainly sponsored by the European Union (FP7) through the ASPRE project (#601852) and Hananja ehf. Hylabs are thanked for providing the PP13 for this study. The University of Vermont is thanked for their internal funding for years 2015 and 2016 to support this work. None of the funding entities had any involvement in the study design or conduct or the interpretation of the results.

## Conflict of interest

Hamutal Meiri and Sveinbjorn Gizurarson are holding patent rights for the use of Pp13 to fight PE. All other author declare no conflict of interest.
